# Estimation of Free-Living Energy Expenditure by Heart Rate and Movement Sensing: A Doubly-Labelled Water Study

**DOI:** 10.1371/journal.pone.0137206

**Published:** 2015-09-08

**Authors:** Søren Brage, Kate Westgate, Paul W. Franks, Oliver Stegle, Antony Wright, Ulf Ekelund, Nicholas J. Wareham

**Affiliations:** 1 MRC Epidemiology Unit, University of Cambridge, Cambridge, United Kingdom; 2 Department of Clinical Sciences, Lund University, Malmö, Sweden; 3 Department of Physics, University of Cambridge, Cambridge, United Kingdom; 4 European Molecular Biology Laboratory, European Bioinformatics Institute, Hinxton, Cambridge, United Kingdom; 5 MRC Human Nutrition Research, Cambridge, United Kingdom; CNRS, FRANCE

## Abstract

**Background:**

Accurate assessment of energy expenditure (EE) is important for the study of energy balance and metabolic disorders. Combined heart rate (HR) and acceleration (ACC) sensing may increase precision of physical activity EE (PAEE) which is the most variable component of total EE (TEE).

**Objective:**

To evaluate estimates of EE using ACC and HR data with or without individual calibration against doubly-labelled water (DLW) estimates of EE.

**Design:**

23 women and 23 men (22–55 yrs, 48–104 kg, 8–46%body fat) underwent 45-min resting EE (REE) measurement and completed a 20-min treadmill test, an 8-min step test, and a 3-min walk test for individual calibration. ACC and HR were monitored and TEE measured over 14 days using DLW. Diet-induced thermogenesis (DIT) was calculated from food-frequency questionnaire. PAEE (TEE ÷ REE ÷ DIT) and TEE were compared to estimates from ACC and HR using bias, root mean square error (RMSE), and correlation statistics.

**Results:**

Mean(SD) measured PAEE and TEE were 66(25) kJ·day^-1^·kg^-1^, and 12(2.6) MJ·day^-1^, respectively. Estimated PAEE from ACC was 54(15) kJ·day^-1^·kg^-1^ (p<0.001), with RMSE 24 kJ·day^-1^·kg^-1^ and correlation r = 0.52. PAEE estimated from HR and ACC+HR with treadmill calibration were 67(42) and 69(25) kJ·day^-1^·kg^-1^ (bias non-significant), with RMSE 34 and 20 kJ·day^-1^·kg^-1^ and correlations r = 0.58 and r = 0.67, respectively. Similar results were obtained with step-calibrated and walk-calibrated models, whereas non-calibrated models were less precise (RMSE: 37 and 24 kJ·day^-1^·kg^-1^, r = 0.40 and r = 0.55). TEE models also had high validity, with biases <5%, and correlations r = 0.71 (ACC), r = 0.66–0.76 (HR), and r = 0.76–0.83 (ACC+HR).

**Conclusions:**

Both accelerometry and heart rate may be used to estimate EE in adult European men and women, with improved precision if combined and if heart rate is individually calibrated.

## Introduction

Precise assessment of energy expenditure (EE) is essential for studies of energy balance and has been used to identify underreporting of food intake [1]. Physical activity energy expenditure (PAEE) is the most variable component of total energy expenditure (TEE) and the most difficult to assess during free-living conditions. The most accurate method is doubly-labelled water (DLW) assessment of TEE combined with measurement of the resting metabolic rate (RMR) via indirect calorimetry [2,3]. These procedures require high levels of technical expertise, are relatively expensive, and yield no information about underlying patterns of energy expenditure over shorter periods of time, e.g. the intensity profile. Wearable sensors have potential to provide estimates of PAEE which may be combined with estimates of REE; an approach which holds promise for better quantifying dose-response relationships between EE and health outcomes, as well as assessing intervention effects in trials, where self-report measures could be biased [4].

For the assessment of PAEE, previous studies have demonstrated the advantage of integrating physiological measures, like heart rate (HR), with biomechanical measures such as acceleration (ACC) or other motion sensing [5–12]. In particular, combining HR and ACC by branched equation modelling may be advantageous for estimating PAEE of structured common daily activities [13–15], unstructured activities [16,17], and a mixture of structured and unstructured activities performed in a whole-body calorimeter over a full day [18]. This technique also allows incorporation of individual calibration information, e.g. in the form of a graded exercise test, with some evidence suggesting PAEE estimates from combined sensing may retain some accuracy even if dynamic individual calibration information was lacking [13,16,18,19].

Most of these studies were conducted under controlled laboratory conditions and only four studies in adults have evaluated combined ACC+HR measures to predict EE during free-living assessed with the DLW technique [10,12,19,20], one only in men and two smaller studies using multiple sensors [10] which may increase the Hawthorne effect [21,22] and may be less feasible for use in large-scale studies. One of these used a uniaxial accelerometer and a heart rate monitor, both calibrated to each of eight individuals’ energy expenditure during treadmill walking and running, and found best precision for combined ACC+HR model estimates, although not significantly different from either of the two single-signal models [12]. Recently, we used a single-piece combined ACC and HR monitor [11], combined with a simple step test for individual calibration [13], to estimate PAEE of urban and rural Cameroonians with reasonable accuracy and precision, higher for individually calibrated estimates [19]. This was replicated in French men using a bike test for individual calibration [20] but no study has so far reported on validity of PAEE or TEE estimates in European women and men and directly compared validity on multiple levels of individual calibration (representing different levels of feasibility).

The purpose of this study was to assess the absolute validity of PAEE and TEE estimates from ACC and HR data at five levels of individual calibration, against free-living EE estimates based on DLW.

## Methods

### Participants

A total of 51 men and women were recruited from the Cambridge area, United Kingdom. Ethical approval for the study was obtained from Cambridgeshire Health Authority Local Research Ethics Committee (LREC03/139). All participants provided written informed consent.

Participants were asked to refrain from eating, drinking (except water), smoking, and vigorous exercise for at least two hours prior to arriving at the laboratory. Following consent, height and weight were assessed using standard anthropometric techniques. Body composition was assessed with the four-compartment model [23] using dual X-ray absorptiometry (Lunar Prodigy, GE Healthcare, USA), air-displacement plethysmography (BodPod, Life Measurement, Inc., USA), and heavy isotope dilution.

### Individual calibration

Approximately an hour after arrival, individual calibration procedures commenced, details of which are described elsewhere [13]. Briefly, resting metabolic rate (RMR) was measured for 45 minutes through indirect calorimetry using a ventilated hood (Oxycon Pro, Jaeger Gmbh, Germany). This calorimetry system has been shown to provide valid measures of ventilation and gas fractions [24], which we confirmed for our specific piece of equipment using mechanical ventilation (iron lung) to dilute known concentrations of bottled oxygen, carbon dioxide, and nitrogen. Participants then underwent an 8-min ramped step test followed by 90-sec recovery, and a ramped treadmill test lasting 20.5 minutes or less if 90% of age-predicted maximal HR [25] was reached. The 3^rd^ min of the treadmill test was used as a separate calibration level, termed ‘walk test calibration’, and data from 4^th^ minute onwards were used for two other treadmill calibration levels (one utilising and one not utilising individual-level calorimetry information) [13]. Total metabolic rate was measured throughout the calibration tests using a face mask interface with the indirect calorimetry system described above. Instantaneous PAEE was defined as total metabolic rate minus RMR. Heart rate and acceleration were measured in all tests with a combined sensor (Actiheart, CamNtech Ltd, Papworth, UK), attached to standard ECG electrodes (Red Dot 2570, 3M) at the level just below the apex of the sternum as described elsewhere [11,26]. The accelerometer in the combined sensor provides a linear (R^2^ = 0.99) measure of acceleration as evaluated during mechanically standardised conditions, and the ECG circuitry in the device provides heart rate measurements which agree with 3-lead ECG values within 4 bpm during clinical tests of rest and treadmill walking and running [11].

Five levels of individual calibration of the HR-PAEE relationship were evaluated in this study (treadmill+VO_2_, treadmill, walk, step, no-exercise [group]); all used net HR (HR above Sleeping heart rate, HRaS) as the quantitative basis [13]. In order to compute complete calibration curves, HR-PAEE relationships were linearly extrapolated downwards to a flex HRaS point [27,28], below which they were extrapolated linearly to resting HR and PAEE = 0 J·min^-1^·kg^-1^. Group-calibrated HR estimates were derived using the average treadmill response from all individuals in the sample as previously described [13], and as a sensitivity analysis an out-of-sample group calibration was derived on the basis of 3865 step tests performed by 1941 adults from 10 European countries [29].

Only one group-calibrated ACC-PAEE equation was evaluated [13], as it has previously been shown that individual calibration of this relationship provides little or no improvement in estimation accuracy for daily living activities [18,30–32].

### Assessment of free-living energy expenditure

Free-living energy expenditure was assessed with the doubly-labelled water (DLW) technique, which is the best standard for assessing TEE during free-living conditions and also compares favourably against whole-body calorimetry [2,3]. Following two baseline urine samples, participants were given a body-weight specific dose of DLW (174mg of H_2_^18^O and 70mg of ^2^H_2_O per kg) and then collected daily urine samples for the next 14 days. Participants were instructed to collect any sample but the first void after getting out of bed, record the time of each sample on both the sampling bottle and a separate recording sheet, and then keep all samples refrigerated until collection by the research team at the end of the 14-day study period. At this time, the European Prospective Investigation into Cancer food frequency questionnaire (FFQ, containing 131 lines) was administered [33]. Isotopic enrichment of all samples was analysed in duplicate with minor modifications to procedures described in detail elsewhere [34]. Briefly, for ^2^H/^1^H ratios 0.4 ml sample was equilibrated with 3 ml H_2_ gas at 1 bar, at 22°C for 6 hours in the presence of a Pt catalyst. The equilibrated gas samples were then measured relative to tank H_2_ in a dual-inlet isotope ratio mass spectrometer (Sira 10, Micromass, Wythenshaw, UK) and enrichment values calculated on the Vienna Standard Mean Ocean Water (SMOW)/ Standard Light Arctic Precipitation (SLAP) scale from laboratory references (traceable to international standards) equilibrated in the same way. For ^18^O, 0.5 ml samples were equilibrated with 10 ml 5% CO_2_ in N_2_ at 1 bar at room temperature, overnight. Ratio measurements were made using an AP2003 continuous flow isotope ratio mass spectrometer (Analytical Precision Ltd, Northwich, Cheshire, UK) and enrichment values calculated on the SMOW/SLAP scale as described for the ^2^H data.

Rate constants for isotope disappearance were estimated using data from all post-dose samples collected over the 14-day period, after baseline correction using the mean of pre-dose samples. If the rate of ^18^O disappearance exceeded 3 half-lives in this time, only samples up to the 3 half-lives time point were used in calculations to maintain equivalence in precision throughout the study [35]. Isotope dilution spaces were calculated from the zero-time intercepts of the isotope disappearance curves. Average internal error of uncorrected CO_2_ production measurements was 5%, as described in detail elsewhere [36,37].

CO_2_ production was calculated using the Schoeller method, which standardises the ^2^H / ^18^O space ratio to 1.04 / 1.01 = 1.03 [38,39]. Criterion total energy expenditure (TEE, in MJ·day^-1^) was calculated according to the Weir equation [40] using the food quotient calculated from the FFQ as a proxy for the respiratory quotient to estimate oxygen consumption. Total daily resting energy expenditure (REE) was calculated as REE = (16 + 8·0.95)/24·RMR to account for sleeping metabolic rate being on average 5% lower than measured RMR (awake, ventilated hood) and assuming 8hrs sleep per 24hrs [41]. Diet-induced thermogenesis (DIT) was calculated from the macro-nutrient energy intake composition of the diet [42] as assessed by the FFQ but normalised by DLW-based TEE / FFQ-based TEE. Criterion PAEE was calculated as TEE ÷ REE ÷ DIT and expressed in kJ·day^-1^·kg^-1^.

### Acceleration and heart rate monitoring during free-living

During the free-living observation period, participants wore the same combined ACC and HR sensor as described for the calibration tests. Participants were supplied with ten extra sets of electrodes and asked to wear the monitor at all times including during showering and swimming. Participants were advised to change their electrodes every second day, in conjunction with taking a shower. Monitors were set up to start data collection at 06:00 the morning following dosing and record minute-by-minute acceleration, trimmed average heart rate, two fastest and two slowest heart beats (inter-beat intervals), average ECG voltage level, and the fraction of time during which the monitor firmware could not detect HR [11]. At these settings, 11.3 days of data could be collected given battery and memory capacity of the monitor.

### Estimation of free-living energy expenditure

HR data were pre-processed using a Bayesian approach as described elsewhere [43]. Briefly, auxiliary variables (HR signal indicator, fastest and slowest heart beats) were used to assign data points to noise clusters, following which Gaussian Process Robust regression incorporating short- and long-term (circadian) covariance functions (priors) was used to infer the true latent HR time-series along with uncertainty estimates. ACC data were checked for anomalies (baseline bleed, axis freezing) but as none occurred in this dataset, these data were analysed in their raw form. Segments of data with continuous zero acceleration lasting >90 minutes were treated as ‘monitor not worn’ if also accompanied by non-physiological HR data (large and prolonged heart rate uncertainty).

PAEE (in kJ·day^-1^·kg^-1^) was calculated by time-integration of the activity intensity (in J·min^-1^·kg^-1^) time-series, estimated from HR and ACC separately and combined ACC+HR in a branched equation model [13,18]. We accounted for any potential diurnal imbalance of wear time by weighting all hours of the day equally in the summation [44].

For single-signal HR estimates, the flex-HR method as described elsewhere [27,28] was used. Briefly, this method translates HR to EE estimates according to an individually established calibration (as described above for each of the five levels) but only for time points where HR is above an individually determined flex point (flex HRaS); below this point activity intensity is estimated to be 0 J·min^-1^·kg^-1^). Specifically for this study, we used a flex HRaS of 10bpm +50% of lowest exercise HRaS, defined as lowest HRaS after 2-min of walking at 3.2 km·hr^-1^ for treadmill and walk-calibrated models and 80% of the 2-min HRaS value while stepping for step calibrated models; the latter is roughly equivalent to the HRaS value after 1-min of stepping but easier to determine reliably, and also comparable to the level of exertion used to define flex HR for the treadmill test. For non-exercise (group) calibrated models, predicted flex HRaS from sleeping HR was used [13].

The branched equation modelling technique assigns different weightings to the HR-PAEE and ACC-PAEE relationships, depending on the epoch-by-epoch observed values [13,18]. Weightings for the two most extreme branches vary slightly between previous evaluations [14,16,18,19]; as this matters most for the lower branch, we consolidated the two weightings, 0% and 10%, in the current study by applying the 0% weighting when average acceleration for the previous 2 min was lower than the movement (“X”) branching point, otherwise the 10% weighting was used.

Estimates of total energy expenditure (TEE, in MJ·day^-1^) were calculated from each model by multiplying PAEE by body weight, adding resting energy expenditure, and dividing this sum by 0.9 to account for diet-induced thermogenesis [45]. For the two models which use individual-level indirect calorimetry in the dynamic calibration, measured RMR from indirect calorimetry (ventilated hood) would be a likely method combination and so this was used to calculate daily REE; for all remaining models, predicted RMR [46] was used to calculate daily REE.

### Statistics

Agreement (absolute validity) between estimated and DLW-measured EE components was assessed with the Bland-Altman technique [47] which allows graphical exploration of method differences and 95% limits of agreement (LoA) across the measurement range. Mean bias was determined by paired t-test. Estimation error (precision) was quantified by the root mean square error (RMSE), ie the square-root of the mean squared differences between methods. In addition, degree of shared variance between model estimates and DLW measures was assessed with Pearson correlation. All recordings with ≥4 days of wear data were included in analyses but each record weighted by the degree of time overlap between the monitor wear and DLW measurements (calculated as monitor wear time / DLW time). Sensitivity analyses were conducted disregarding measured RMR in all calculations (estimation and criterion), and instead using predicted RMR calculated with both the Oxford equations [46] and the Schofield equations [48].

All analyses were performed in STATA 13.1 SE (StataCorp, TX, USA), except the Gaussian Process Regression which was done in JAVA with a MySQL database. Programs and syntax files are available from www.mrc-epid.cam.ac.uk/research/resources.

## Results

### Data completeness

Free-living energy expenditure could be calculated from DLW in all but one individual (missing timed urine samples). Free-living ACC+HR data were complete (11 days of data) in 44 individuals (n = 43 with DLW results). The remaining 7 individuals had incomplete data as follows: We were unable to download data from one individual (100% missing data); and in five individuals, two recordings ended after <2.5 days (excluded), and three recordings were 4–6 days in length (included). In one individual, acceleration data were available for all 11 days but no heart rate signal was recorded; this individual is also excluded from all analyses. Therefore, 46 individuals contributed data to the present analyses, characteristics of whom are shown in Table 1.

**Table 1:** Participant characteristics.

	Women			Men		
	*n = 23*			*n = 23*		
	Mean	SD	Range	Mean	SD	Range
Age (yrs)	35.0	(10.1)	22–55	33.2	(8.1)	23–48
Weight (kg)	62.8	(9.2)	48–81	78.2	(12.7)*	53–104
Height (m)	1.62	(.06)	1.50–1.75	1.77	(.07)*	1.67–1.89
BMI (kg·m^-2^)	24.1	(3.8)	19–34	24.9	(3.2)	19–32
Body fat (%)	32.6	(7.7)	15–46	21.9	(8.2)*	8–35
VO_2_max (ml O_2_·min^-1^·kg^-1^)	34.8	(8.9)	22–56	45.0	(11.5)*	25–67
Food quotient	0.87	(0.2)	0.84–0.93	0.86	(0.2)	0.80–0.90
TEE (MJ·day^-1^)	10.3	(1.6)	7.9–13.6	13.7	(2.3)*	8.2–19.2
REE (MJ·day^-1^)	5.4	(0.6)	4.4–6.7	7.0	(1.0)*	4.5–8.9
REE (kJ·day^-1^·kg^-1^)	87.2	(8.9)	70–101	90.4	(11.8)	69–116
PAEE (kJ·day^-1^·kg^-1^)	62.0	(21)	21–99	70.3	(26)	28–130
PAL	1.91	(.30)	1.36–2.42	1.97	(.30)	1.49–2.55

BMI, Body Mass Index; VO_2_max, Maximal oxygen uptake (estimated from individual HR-VO_2_ relationship during treadmill test); REE, Resting Energy Expenditure; TEE, Total Energy Expenditure; PAEE, Physical Activity Energy Expenditure; PAL, Physical Activity Level (TEE/REE). Mean (SD) isotopic space ratio was 1.037 (0.01).

*p<0.05 different from women.

### Measured energy expenditure

Mean (SD) TEE for the whole sample was 12.0 (2.6) MJ·day^-1^ or 172 (31) kJ·day^-1^·kg^-1^, REE was 6.2 (1.2) MJ·day^-1^ or 89 (10) kJ·day^-1^·kg^-1^, and DIT was 9.9 (0.9) % of TEE, resulting in an average physical activity level (TEE/REE) of 1.94 (.30) and a PAEE of 66.1 (24) kJ·day^-1^·kg^-1^ (Table 1). There were no significant differences between men and women with respect to energy expenditure when normalised by body weight but women had significantly higher water turnover (p<0.01).

Predicted RMR from the Oxford equations [46] was not significantly different from measured RMR values (prediction bias: +1.2 J·min^-1^·kg^-1^ or 1.9%; p = 0.15, 95%LoA-11; 13 J·min^-1^·kg^-1^); however predicted RMR using the Schofield equations [48] was positively biased (prediction bias: +5.8 J·min^-1^·kg^-1^ or 9.3%; p<0.001, 95%LoA-7; 19 J·min^-1^·kg^-1^), and corresponding PAL therefore lower at a mean of 1.77. Even so, results from sensitivity analyses using either of the two predicted estimates of RMR instead of measured values were largely similar (S1 Table); hence only results using measured RMR for calculating criterion values are presented below.

**Fig 1:**
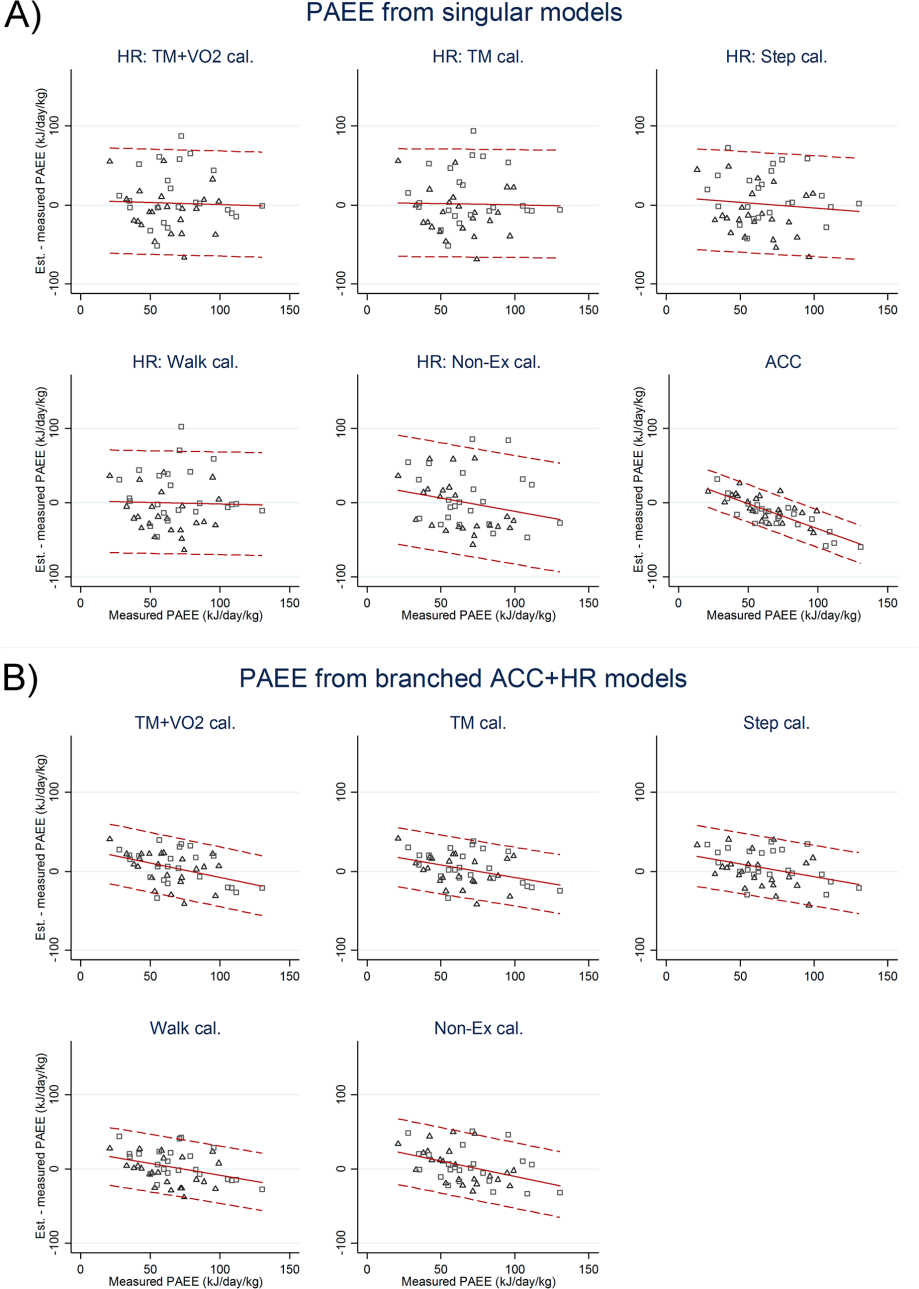
Physical Activity Energy Expenditure (Bland-Altman plot). Difference between DLW-measured and heart rate and accelerometry estimated physical activity energy expenditure (PAEE) in kilojoules per kg per day for adult women (triangles) and men (squares) plotted against DLW-measured PAEE. Broken lines are mean (±2 SD) estimation errors.

**Fig 2:**
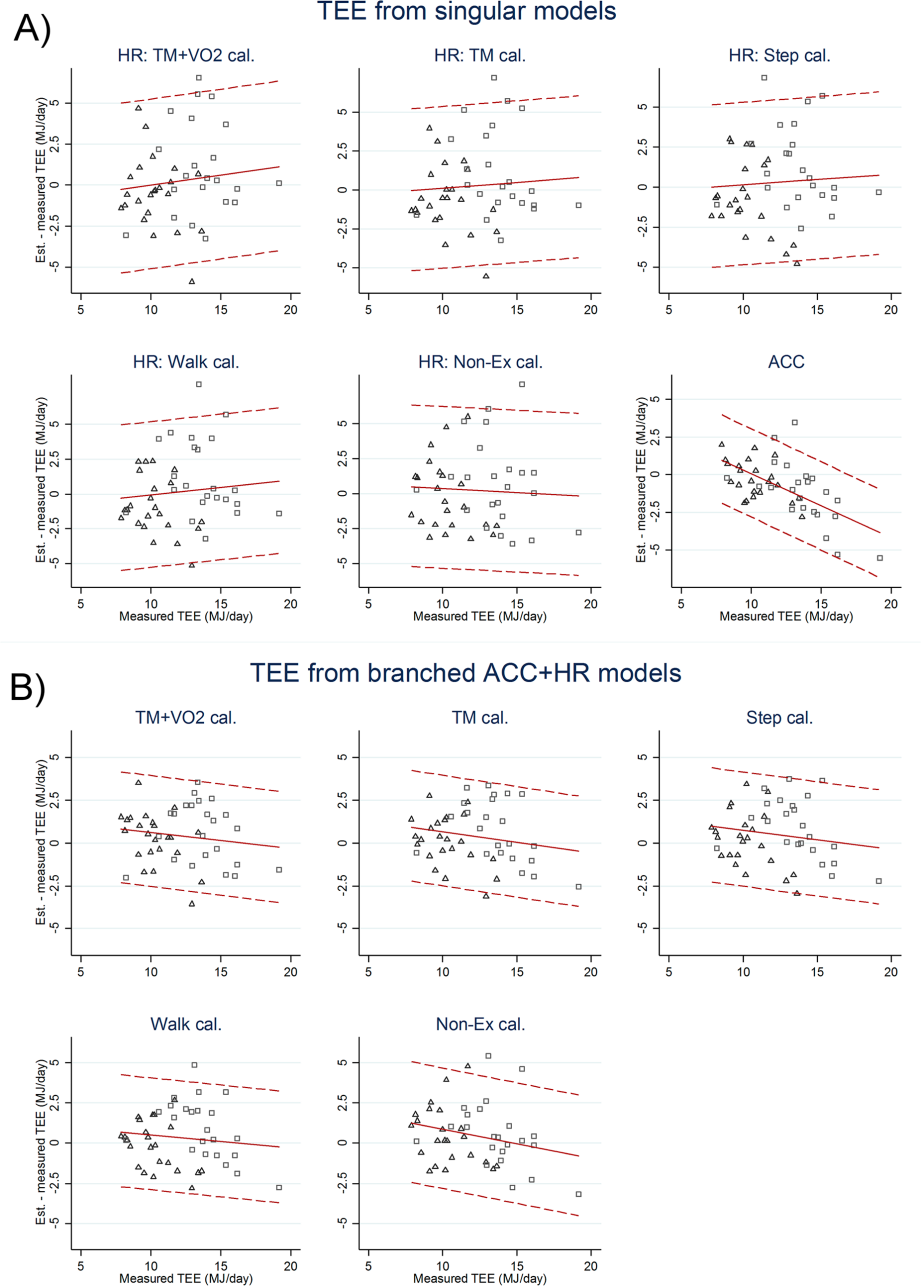
Total Energy Expenditure (Bland-Altman plot). Difference between DLW-measured and heart rate and accelerometry estimated total energy expenditure (TEE) in Megajoules per day for adult women (triangles) and men (squares) plotted against DLW-measured TEE. Broken lines are mean (±2 SD) estimation errors.

For physical activity research, the agreement analyses of PAEE (Table 2, Fig 1) are the most relevant, whereas TEE results (Table 2, Fig 2) are likely more applicable for research on energy balance.

**Table 2:** Validity of ACC-, HR-, and combined ACC+HR equations for predicting Energy Expenditure.

		Physical Activity Energy Expenditure				Total Energy Expenditure			
Model	*Individual calibration*	Mean (SD)	Bias (RMSE)	95% LoA	r	Mean (SD)	Bias (RMSE)	95% LoA	r
DLW		66.1 (24)				12.0 (2.6)			
ACC	*No exercise test*	53.7 (15)	-12.1*(24)	-53; 29	.52	11.2 (2.1)	-0.78*(1.99)	-4.4; 2.8	.71
Flex HR	*TM + VO*_*2*_	68.6 (41)	3.5 (33)	-61; 68	.58	12.2 (3.9)	0.32 (2.57)	-4.7; 5.4	.76
Flex HR	*TM*	67.4 (42)	2.3 (34)	-64; 69	.58	12.3 (3.8)	0.32 (2.56)	-4.7; 5.4	.74
Flex HR	*Step*	67.2 (39)	2.4 (32)	-60; 65	.57	12.3 (3.8)	0.37 (2.51)	-4.6; 5.3	.75
Flex HR	*Walk*	65.7 (42)	0.2 (34)	-67; 67	.57	12.1 (3.9)	0.19 (2.58)	-4.9; 5.3	.75
Flex HR	*No exercise test*	66.9 (39)	1.9 (37^#^)	-71; 75	.40	12.2 (3.8)	0.34 (2.85^#^)	-5.3; 5.9	.66
Branch ACC+HR	*TM + VO*_*2*_	70.7 (25)	5.5 (21^§^)	-35; 46	.64	12.4 (2.9)	0.49*(1.68^§^)	-2.7; 3.7	.83
Branch ACC+HR	*TM*	69.0 (25)	3.8 (20^§^)	-35; 43	.67	12.4 (2.8)	0.45 (1.68^§^)	-2.7; 3.7	.82
Branch ACC+HR	*Step*	70.3 (25)	5.2 (21^§^)	-35; 46	.66	12.5 (2.9)	0.57*(1.75^§^)	-2.7; 3.9	.82
Branch ACC+HR	*Walk*	68.2 (26)	2.8 (21^§^)	-38; 43	.66	12.4 (3.0)	0.40 (1.76^§^)	-3.0; 3.8	.81
Branch ACC+HR	*No exercise test*	70.3 (26)	5.1 (24^§^)	-42; 52	.55	12.5 (2.8)	0.55 (1.97^§^)	-3.2; 4.3	.76

PAEE: Physical Activity Energy Expenditure (kilojoules·day^-1^·kg^-1^), TEE: Total Energy Expenditure (Megajoules·day^-1^), RMSE: Root Mean Square Error, LoA: Limits of Agreement, DLW: Doubly-labelled Water; ACC: Acceleration; HR: Heart Rate; TM: Treadmill test; VO_2_: Oxygen consumption response measured.

*Different from measured PAEE or TEE (p<0.05)

^#^different from ACC estimate (p<0.05)

^§^different from corresponding HR estimate on same calibration level (p<0.05).

Mean(SD) values weighted equally across the 46 included individuals; agreement and correlation analysis weighted by degree of time overlap between monitor wear and DLW measurements.

### Accelerometry-estimated energy expenditure

All ACC values observed were in the 0–10 m·s^-2^ range. As shown in Table 2, mean PAEE estimated from the ACC model was on average 28% lower than the DLW-measured values (p<0.001), and the standard deviation (SD) was about 60% of the SD of DLW-measured PAEE. However, the two methods were moderately correlated (r = 0.52, p<0.01).

Estimation errors were strongly negatively correlated with level of activity (r = -0.80, p<0.001) in such a way that PAEE of the least active individuals (<50 kJ·day^-1^·kg^-1^) agreed reasonably well with DLW-measured values whereas PAEE of more active individuals was progressively underestimated (Bland-Altman plots, Fig 1). Estimation errors were also correlated with body fat percentage (r = 0.63, p = 0.001) which explained an apparent difference between men and women.

For total energy expenditure, the underestimation of the ACC model combined with predicted REE amounted to about 6% of TEE (p<0.001) but estimates were highly correlated (r = 0.71) with DLW-measured TEE (Table 2). Estimation errors displayed a negative correlation (r = -0.62, p<0.001) with DLW-measured TEE (Bland-Altman plots, Fig 2), similar to that observed for PAEE. Average estimation errors (RMSE) for PAEE and TEE were 36% and 17% of average measured values (27% and 32% of REE), respectively.

### Flex-HR estimated energy expenditure

Mean(SD) flex HR was 75.9(11) bpm using treadmill exercise and similar for step test. Approximately 62% of free-living time was spent below the flex-HR point. PAEE and TEE from flex-HR models were not significantly different from corresponding measures from DLW but the between-individual variance (SD) in HR-estimated PAEE was approximately twice as high as the SD of DLW-measured PAEE (Table 2). Estimation errors were not significantly associated with DLW-measured energy expenditure (Bland-Altman plots, Figs 1 and 2), nor were they related to body fat percentage and sex, except for the out-of-sample model (inverse association with EE and positive association with fatness and male sex). Average estimation error (RMSE values) was about 50% of average PAEE (37% of REE) across all levels of dynamic individual calibration and slightly higher for the model with no exercise calibration; only RMSE of the latter was significantly higher than the RMSE for the ACC model (p<0.001). Correlations between flex-HR estimates and DLW-measured values were moderate-to-high for models using some form of dynamic calibration (r~0.58 for PAEE and r~0.75 for TEE) but only modest for the non-exercise calibrated model. The out-of-sample model (also without dynamic calibration) was even less precise, with non-significant bias (RMSE) of-5.3 (35) kJ·day^-1^·kg^-1^ and weaker correlation (r = 0.18) for PAEE (other results not shown).

### Branched ACC+HR model estimated energy expenditure

Heart rate and acceleration data collected during free-living were distributed such that heart rate was effectively utilised 25% of the time in the branched equation model. Both means and SD values of estimated PAEE from branched ACC+HR models were not dissimilar to those for DLW-measured PAEE and TEE for all levels of individual calibration, although mean TEE estimates were about 0.5 MJ·day^-1^ (4%) higher than measured TEE (Table 2). All correlations were moderate-to-high for PAEE (r = 0.55–0.67) and high for TEE estimates (r = 0.76–0.83).

Estimation errors were inversely (p≤0.003) associated with energy expenditure (Bland-Altman plots, Figs 1 and 2) but not related to body fat percentage except for the out-of-sample model (r = 0.77, p<0.001). Estimation precision (RMSE) of branched models was not significantly different between levels of individual calibration at around 32% of measured PAEE and 14% of measured TEE (both roughly 25% of REE), nor were they significantly different from the single-signal ACC model but all combined models were significantly more precise than their corresponding flex-HR models on the same level of individual calibration. The out-of-sample model was the least precise, with biases (RMSE) of-0.2 (24) kJ·day^-1^·kg^-1^ and-0.2 (2.1) MJ·day^-1^ and correlations r = 0.37 and r = 0.68 for PAEE and TEE, respectively.

## Discussion

We report here on absolute and relative validity of PAEE and TEE estimates from ACC and HR monitoring in a UK sample of adult men and women who varied greatly in age, fitness, body composition, and level of physical activity, with the most active individual spending about five times as much energy on physical activity as the least active for both genders. Our main findings were that the non-individualised locomotion-based accelerometry equation underestimated free-living PAEE by about 24% with error structure related to body composition, whereas flex-HR and combined ACC+HR models could estimate PAEE with no mean bias, regardless of calibration level and with error being random with respect to fatness. Precision (RMSE) was best for combined ACC+HR models with some dynamic calibration; the difference in precision to other models was significant when compared to the single-signal HR models but not when compared to the single-signal accelerometry model. Relative validity (correlations) was generally moderate-to-high for all models.

### Accelerometry-estimated energy expenditure

PAEE was underestimated by the ACC model in most individuals, and with errors showing a relatively strong correlation with body fat percentage. This inter-relationship between accelerometry, body composition, and energy expenditure has also been observed by Masse et al [49], which warrants some caution when interpreting associations between the accelerometry estimate of PAEE and metabolic health outcomes. All observed ACC values were well below the maximal value that can be stored in the Actiheart memory (31·2^7^ = 3968 counts), making bias by data storage truncation unlikely. For an epoch setting of 1 minute, this maximal value corresponds to 12m·s^-2^ [11]. By contrast, Bouten and colleagues found it necessary to exclude all recorded accelerations above approximately 8 m·s^-2^ (~0.8*G*) from a waist-worn tri-axial accelerometer (first-generation Tracmor). This was done under the assumption that accelerations >8 m·s^-2^ result from non-human movement, e.g., vibrations from passive transportation (bus, motorbike, sitting on the back of a bike, etc.), and was cross-checked against individual activity logs [30]. Although an 8 m·s^-2^ threshold would filter out some running activity [50], it may have been necessary for the specific accelerometer used by these investigators; a piezo-resistive accelerometer with a bandwidth filter of 0.11–20Hz. The accelerometer in Actiheart is of the piezo-electric type, incorporating filtering for movement frequencies above 7Hz (3db attenuation), resulting in high linearity with acceleration in the 0–4Hz range [11]. In addition, measuring acceleration along the longitudinal axis of the trunk may be less prone to high-frequency noise than measurements from hip or waist because human tissue generally absorbs vibration. Nonetheless, our accelerometry observations agree with those of Leenders and colleagues who found 7-day free-living PAEE to be underestimated from waist-worn motion sensors, including uni- and tri-axial accelerometers and a pedometer [51]. Between 18 and 30% of the variance in PAEE was explained by motion sensing in that study, which is comparable to the 27%, 29% and 22% for the ACC model in the present study, Cameroon, and France, respectively [19,20]. This is also similar to 29% and 34% added variance from uniaxial and triaxial acceleration in a sample of Dutch twins, although the latter was reported to be as high as 60%, depending on the statistical model chosen [52]; however these results have not yet been replicated in other populations. In all studies evaluating absolute validity, a reduced between-individual variance in motion sensor estimates of PAEE was observed. Such regression bias towards the mean in the exposure variable may lead to an overestimation of the dose-response relationship between predicted physical activity energy expenditure and health outcomes, which contrasts the effect of measurement error from HR models [53,54]. Thus, conclusions on dose-response relationships involving physical activity should be made with caution if PAEE is estimated from single-signal ACC or HR models, although other sources of error also come into play [55].

### Flex-HR estimated energy expenditure

For single-signal flex-HR models, mean bias for estimating PAEE was non-significant for all; however RMSE and correlations were around 33 kJ·day^-1^·kg^-1^ and 0.57, respectively, for all models using some form of exercise calibration, and worse for models with no dynamic calibration at the individual level. Part of the explanation for these differences is related to the definition of flex-HR, which in this study was calculated as 10bpm + 50% of the observed HRaS during slow walking or gentle stepping or predicted from sleeping HR. In the original flex-HR studies using whole-body calorimetry as the criterion measure, flex-HR was defined as the average between highest HR during rest (lying awake, sitting, and standing) and lowest HR during activity [27,28], averaging around 81bpm but based on *post hoc* analysis another 10 bpm was added to correct for a small but significant overestimation of the group estimate of TEE; our estimates using an offset of 10bpm but using sleeping HR as the resting point to increase feasibility (less testing required) are closer to the *a priori* definition with a mean around 76 bpm in this sample which is also comparable to larger population samples [56,57]. However, it should be noted that the flex HR point does not perfectly discriminate activity from rest, and typically fails to remove all influence of non-activity related elevations in HR and on other occasions filter out real low-intensity activity which may still be relevant for health [54,58]. Nonetheless, our results for daily EE estimation are in close agreement with those reported by Livingstone and colleagues using estimates of EE from 2–4 days HR-monitoring against DLW over 14 days [59], with RMSE = 34 kJ·day^-1^·kg^-1^ and r = 0.6 for PAEE. An inflation of the between-individual variance of flex-HR estimates compared to DLW estimates was also observed, again similar to our results and those reported in Japanese [60,61] and French [20] but in contrast to the observations from Cameroon [19] and a small sample from Sweden [12]. Some of this inflation may, however, be explained by discrepancies in length of HR and DLW monitoring periods, as Davidson and colleagues observed only a small (12%) inflation of variance in nine adult men following a 9-day monitoring protocol for both HR and DLW after accounting for error in DLW estimates [62]. These investigators did, however, also use an extensive individual calibration protocol, utilising both 27-hour whole-body calorimetry and additional high-intensity exercises and were able to obtain a slightly higher PAEE precision (RMSE = 32 kJ·day^-1^·kg^-1^, r = 0.8) after HR data had been cleaned and missing data imputed, a process which was shown to profoundly improve EE estimates. Finally, as heart rate also increases after eating [63,64], this may or may not be reflected in the estimate of energy expenditure, depending on whether heart rate was above the flex HR point or not; this is because all observations below flex HR would be translated as resting metabolic rate. About a third of observations were above flex HR in this study; if we assume the same 2:1 distribution of time in the postprandial phase following three meals per day and that meals would increase heart rate by an average of 10bpm [64] and stay elevated for two hours after each meal, the theoretical contribution to the PAEE estimates from this source would be in the order of 7 kJ·day^-1^·kg^-1^. This would constitute a positive bias of the PAEE estimates, which, if corrected, suggests that flex-HR model estimates of energy expenditure may be negatively biased; that said this underestimation was not significant for neither PAEE nor TEE in the present study when the data were re-analysed this way.

### Branched ACC+HR model estimated energy expenditure

Combining ACC with HR by branched equation modelling resulted in improved accuracy and precision for estimating PAEE and TEE, with little or no significant bias, and similar between-individual variance (SD) of the PAEE estimates to that observed for DLW. In addition, there was no evidence of differential bias by body fat percentage. The main attraction of this modelling approach is that it has established high validity for estimating activity intensity [14,16], as well as validity of the time-integral (area under the curve) of the activity intensity time-series, ie daily PAEE, as demonstrated in the present study. The RMSE of the PAEE estimates was between 20 and 24 kJ·day^-1^·kg^-1^ with no clear trend across the various levels of dynamic individual calibration but somewhat higher without dynamic calibration. Precision of branched models was higher than precision of single-signal models but only significantly so when compared to the flex-HR models. These results from the UK corroborate our previous findings in Cameroon [19], generally showing higher precision for combined ACC+HR models, with RMSE 37, 35, and 29 kJ·day^-1^·kg^-1^, for ACC, step-calibrated flex-HR, and step-calibrated ACC+HR models respectively, for rural and urban participants analysed together and with estimates being more precise in the urban subsample, e.g. RMSE = 19 kJ·day^-1^·kg^-1^ for the step-calibrated combined ACC+HR model. However, unlike the correlations reported here in the UK sample, the correlations in Cameroon were modest (r~0.4) for combined ACC+HR models and actually highest for the ACC model (r = 0.54). Although precision was reported to be about 30% higher when outliers were excluded, there may be other explanations for these differences; more refined methods for handling sensor noise of the HR signal and potential diurnal information bias was used in the present study [43,44], and there are also small differences in flex-HR definitions and the implementation of the branched modelling technique [13,18]. As for the latter, recent results by Villars et al in 35 French men, recruited by activity and obesity level [20], are likely closer to the implementation of the current study; these authors reported high precision of individually bike-test calibrated PAEE estimates (RMSE of 14 kJ·day^-1^·kg^-1^, r = 0.81), which compared favourably to non-calibrated and single-signal models, e.g. RMSE = 33 kJ·day^-1^·kg^-1^ and r = 0.47 for the ACC-only model. This study also used the less refined method for handling sensor noise but used imputation of missing data by the mean awake or sleep periods as captured by self-report and attempted to limit diurnal information bias by only including days with >80% of awake time being valid. In the current study, we use all valid data available in each record but apply equal weighting to the 24 hours of the day [44]. The French study also included an evaluation of the models’ capability to detect within-individual PAEE change in a sub-sample (n = 21) subjected to an intervention, elegantly demonstrating the benefit of individual calibration, with RMSE of 13 vs 18 vs 30 kJ·day^-1^·kg^-1^ and correlations of r = 0.63 vs r = 0.37 vs r = 0.11, for repeat calibrated vs baseline-only calibrated vs non-individually calibrated models [20]. All in all, it is reasonable to conclude that some degree of dynamic individual calibration, even if only for a sub-sample of the study population, is advisable as the gain in validity should outweigh the loss in feasibility.

Using a different branched modelling technique, Johansson and colleagues also observed higher precision for individually-calibrated combined ACC+HR compared to single-signal ACC models, RMSE 31 vs 34 kJ·day^-1^·kg^-1^, respectively, although this was not significant in their sample of eight Swedish participants [12]. The similarity in precision between combined ACC+HR and estimation based only on ACC was also observed by Hustvedt and co-workers, who did report improvements in ActiReg precision after adding HR to their motion-based model, but only in the most active individuals [10].

As was discussed for flex-HR models, the postprandial effect on heart rate may result in over-estimation of PAEE using fasted exercise calibration equations; for combined ACC+HR model estimates in the present study where HR was effectively utilised 25% of the time, the impact of the same theoretical heart rate elevations and meal patterns as outlined above (+10bpm over 2hrs, three meals/day) would translate into an overestimation of PAEE of about 5 kJ·day^-1^·kg^-1^. If we correct ACC+HR model estimates of PAEE and TEE for this effect, it would almost eliminate the small (non-significant) positive bias of most combined ACC+HR model estimates. It is, however, important to remember that if the postprandial period was predominantly spent at rest, there would be no inflation of the PAEE estimate from the ACC+HR model.

### Strengths and limitations

Although the DLW technique may be considered the gold standard for assessment of energy expenditure during extended periods (weeks) of free-living, the method is not without limitations. Indeed, error in DLW estimates may be as high as 8% for TEE and more for PAEE (up to 20% for a PAL of 2.0 and 40% for a PAL of 1.43) because the analytical error of the technique is absolute. Thus, PAEE of sedentary individuals is measured with greater relative random error than PAEE of active individuals, and the same would be true for individuals with a greater body water-to-body weight ratio (leaner individuals) because DLW is administered on the basis of body weight. Although analytical error is believed to be random and thus tend to inflate observed variance, we limited this error in the present study, firstly by obtaining two baseline urine samples (50% of analytical error may originate from variation in background enrichment), secondly by undertaking duplicate analyses for the isotopic enrichment of each urine sample, thirdly by collecting multiple post-dose urine samples during the measurement period, and fourthly by truncating analysis to samples within 3 half-lives of ^18^O disappearance. Moreover, the standardisation of isotopic dilution spaces reduces inflation of between-individual variance but ignores the biological variance that may exist which could impact on individual TEE and PAEE estimates [35,36,38,65], although measured dilution spaces in the current study were as expected. Finally, the precision of the DLW method used in the present study relies on the accuracy with which the participants report the collection time of urine samples and also on the assumptions on respiratory quotient and DIT which were calculated using data from a subjective food instrument as also done in other DLW studies [51,59]. Given the inherent problems with subjective dietary assessment methods [66–69], an alternative approach would have been to ignore the biological variance in dietary composition and apply the same factors for everybody. It is important to note, however, that DIT does vary depending on the individual’s diet, in particular protein intake [42]. That said, variance in estimated DIT was only 8–13% in the present study which is within the normal range [45]. As mentioned, substrate utilisation, or its proxy measure macronutrient composition of the diet, also influences the conversion of CO_2_ production into TEE and if true respiratory quotient was say 0.8 or 0.9 compared to 0.85, TEE estimates would be about 5% higher or lower, respectively; most of the estimates evaluated here were within that range. A final limitation to the calculation of criterion PAEE pertains to the estimate of REE, which in this study was based on a single 45-min ventilated hood measurement of RMR performed during thermo-neutral laboratory conditions; it is, however, conceivable that this could vary from day-to-day and also deviate at night more or less than the 5% adjustment made. However, as our sensitivity analyses show, validity results were similar when using predicted RMR in all calculations instead of measured RMR, dismissing instability of the RMR measure as a major source of error. Reassuringly, measured RMR was also similar to predicted RMR using the most recent prediction equations [46]. Taken together, the limitations in the criterion measures of PAEE and TEE are unlikely to have materially influenced the conclusions drawn on the validity of the different model estimates using data from the wearable sensor; if anything, validity estimates as presented are likely to be conservative, since any error in the criterion would be attributed to the model estimate and weaken correlations.

Other limitations of the present study include the extent to which data were missing due to monitor non-wear or malfunctioning; the latter may be due to not all monitors being 100% waterproof which was deemed plausible by the manufacturer who has since altered the casing design and upgraded memory capacity 4-fold. Monitor non-wear, however, is likely to also be a characteristic of studies in which the methods are applied but the summation method used in this and other studies [29,70–75] to integrate activity intensity time-series to one value per person minimises the impact of diurnal information bias [44]. That said, the greater extent of missing data from free-living monitoring compared with laboratory conditions will inflate uncertainty and thus partly explains why PAEE assessed using the same method is often closer to the criterion value in laboratory evaluations [11,13,18].

In summary, the locomotion-based ACC model underestimates free-living PAEE, with differential bias to body composition. Both flex-HR and combined ACC+HR models can estimate free-living PAEE with no significant mean bias, irrespective of individual calibration level but precision is higher with some dynamic calibration. Precision was significantly higher for combined ACC+HR models compared to single-signal HR models but not significantly different from precision of the single-signal ACC model. All evaluated models have moderate-to-high relative validity for estimating PAEE and TEE.

## Supporting Information

S1 Table Validity of ACC-, HR-, and combined ACC+HR equations using predicted Resting Metabolic Rate. (DOCX)
