# Supplementary material for: Estimation of Free-Living Energy Expenditure by Heart Rate and Movement Sensing: A Doubly-Labelled Water Study
Source: PLoS One. 2015 Sep 8;10(9):e0137206. doi: 10.1371/journal.pone.0137206 (PMC4562631; doi:10.1371/journal.pone.0137206)
Supplement: S1 Table — (DOCX) [file pone.0137206.s001.docx]

**SUPPLEMENTARY TABLE S1:**

Validity of ACC-, HR-, and combined ACC+HR equations using predicted Resting Metabolic Rate

|  |  |  | Physical Activity Energy Expenditure | | |  | | Total Energy Expenditure | | |  | |
| --- | --- | --- | --- | --- | --- | --- | --- | --- | --- | --- | --- | --- |
| Model | *Individual calibration* | Bias (RMSE) | | 95% LoA | r | | Bias (RMSE) | | 95% LoA | r | |  |
| ACC | *No exercise test* | -12.6^*^(27) | | -59; 34 | .44 | | -0.92^*^(2.04) | | -4.5; 2.7 | .71 | |  |
| Flex HR | *TM + VO_2_* | 3.5 (33) | | -61; 68 | .64 | | 0.32 (2.57) | | -4.7; 5.4 | .76 | |  |
| Flex HR | *TM* | 1.7 (33) | | -63; 66 | .61 | | 0.18 (2.55) | | -4.9; 5.2 | .74 | |  |
| Flex HR | *Step* | 1.9 (31) | | -60; 64 | .57 | | 0.24 (2.49) | | -4.7; 5.2 | .75 | |  |
| Flex HR | *Walk* | -0.4 (33) | | -67; 66 | .58 | | 0.04 (2.56) | | -5.0; 5.1 | .75 | |  |
| Flex HR | *No exercise test* | 1.4 (36) | | -70; 73 | .45 | | 0.20 (2.83) | | -5.4; 5.8 | .65 | |  |
| Branch ACC+HR | *TM + VO_2_* | 5.5 (21^§^) | | -35; 46 | .69 | | 0.49 (1.68^§^) | | -2.7; 3.7 | .83 | |  |
| Branch ACC+HR | *TM* | 3.2 (21^§^) | | -37; 44 | .67 | | 0.32 (1.64^§^) | | -2.9; 3.5 | .82 | |  |
| Branch ACC+HR | *Step* | 4.7 (22^§^) | | -37; 47 | .66 | | 0.44 (1.71^§^) | | -2.9; 3.7 | .82 | |  |
| Branch ACC+HR | *Walk* | 2.2 (22^§^) | | -41; 45 | .64 | | 0.26 (1.72^§^) | | -3.1; 3.6 | .81 | |  |
| Branch ACC+HR | *No exercise test* | 4.6 (24^§^) | | -43; 52 | .58 | | 0.41 (1.93^§^) | | -3.3; 4.2 | .76 | |  |

| PAEE: Physical Activity Energy Expenditure (kilojoules∙day^-1^∙kg^-1^), TEE: Total Energy Expenditure (Megajoules∙day^-1^), RMSE: Root Mean Square Error, LoA: Limits of Agreement, ACC: Acceleration; HR: Heart Rate; TM: Treadmill test; VO_2_: Oxygen consumption response measured.  ^*^Different from DLW-measured PAEE or TEE (p<0.05); ^†^different from No-Exercise-test calibration level (p<0.05); ^#^different from ACC estimate (p<0.05), ^§^different from corresponding HR estimate on same calibration level (p<0.05). Data from 46 individuals.  RMR predicted using equations by Henry CJ (2005): *Basal metabolic rate studies in humans: measurement and development of new equations*. Public Health Nutr 8: 1133-1152. |
| --- |
